# Concurrence of multiple aneurysms, extreme coiling of the extracranial internal carotid artery and ipsilateral persistent primitive hypoglossal artery: A case report and literature review

**DOI:** 10.3389/fneur.2022.1053704

**Published:** 2022-12-05

**Authors:** Zheng Wan, Tianyi Liu, Ning Xu, Qin Liu, Xiaodong Yu, Honglei Wang

**Affiliations:** Department of Neurosurgery, The First Hospital of Jilin University, Changchun, China

**Keywords:** persistent primitive hypoglossal artery, tortuosity of ICA, multiple cerebral aneurysms, congenital vascular anomaly, carotid-basilar anastomoses

## Abstract

**Background:**

The primitive hypoglossal artery (PHA) is an anastomotic vessel of the carotid-basilar artery system that is prevalent only transiently during the embryonic period. Persistent primitive hypoglossal artery (PPHA) is a rare vessel variation in which PHA exists persistently in adulthood and occurs in approximately 0.02–0.1% of the population. Tortuosity of the extracranial internal carotid artery (ICA) is relatively common, impacting 10–43% of the population, and is caused by either congenital or acquired factors. It is still unknown whether PPHA and tortuosity of extracranial ICA are associated. Here, we present a case report of the concurrence of three types of pathologies of the carotid artery: extreme coiling of the extracranial internal carotid artery, multiple aneurysms and persistent primitive hypoglossal artery.

**Case description:**

A 66-year-old woman suffered intermittent headaches, dizziness and numbness of the right eyelid for 5 years. Magnetic resonance angiography performed in a local hospital reported an aneurysm of the posterior communicating artery segment of the left ICA and a left PPHA. Digital subtraction angiography conducted after admission showed a PPHA originating from the left cervical ICA and an extremely coiling segment of the ICA distal to the beginning of PPHA. Except for the aneurysm of the posterior communicating artery segment of the left ICA, multiple aneurysms were found at the coiling segment of the ICA.

**Conclusion:**

To the best of our knowledge, this is the first report of PPHA accompanied by an adjacent, extremely coiling ICA. There are no reports of similar tortuous ICAs to this extent or at this position. Including aneurysms, three types of pathologies suggest their congenital origin, and a review of the literature infers the probable association of these lesions.

## Introduction

The primitive hypoglossal artery (PHA) only exists briefly during early embryonic development, and its function is to supply blood to the posterior circulation from the primitive internal carotid artery before the mature development of the hindbrain vasculature. When PHA fails to degenerate normally and persists into adulthood, this vessel is an anastomotic branch between the internal carotid artery and vertebrobasilar artery (1). It has been reported that PPHA is associated with intracranial vascular anomalies, such as cerebral aneurysms (2–4) and arteriovenous malformations (5, 6). Few cases of PPHA with moyamoya disease have been reported (7). Because of the connection of the ICA and basilar artery by the PPHA, ischaemic stroke in the brainstem or cerebellum occurs when stenosis of the proximal carotid artery exists (8, 9). Morphological anomalies of the external ICA are classified into three types: tortuosity, kinking and coiling. The incidence of these anomalies ranges between 10 and 43% (10). Among the three types of morphological anomalies of the external ICA, it is considered that tortuosity and coiling are congenital, while kinking is an acquired abnormality (11). Since the persistence of PPHA and tortuosity and coiling of the ICA are all congenital vascular variations, there might be an association between PPHA and morphological anomalies of the ICA. Nonetheless, there have been no studies on this topic thus far. Here, we present a case report of PPHA with adjacent extreme coiling of the ICA and multiple aneurysms. The extent of coiling of the ICA is peculiar and has not been reported previously. We also conducted a literature review to discuss the probable relationship of the three vascular pathologies.

## Case description

**Figure 1:**
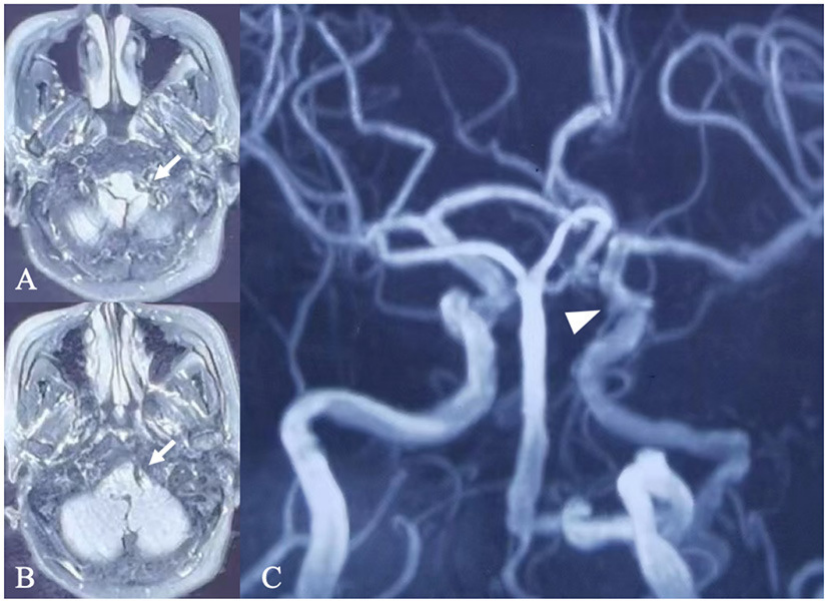
Magnetic resonance imaging and angiography. **(A)** Magnetic resonance imaging **(A,B)** and magnetic resonance angiography **(C)** performed in a local hospital showed a left persistent primitive hypoglossal artery converging into the proximal part of the basilar artery (arrow) and aneurysm of the left posterior communicating artery segment of the ICA (arrow head).

**Figure 2:**
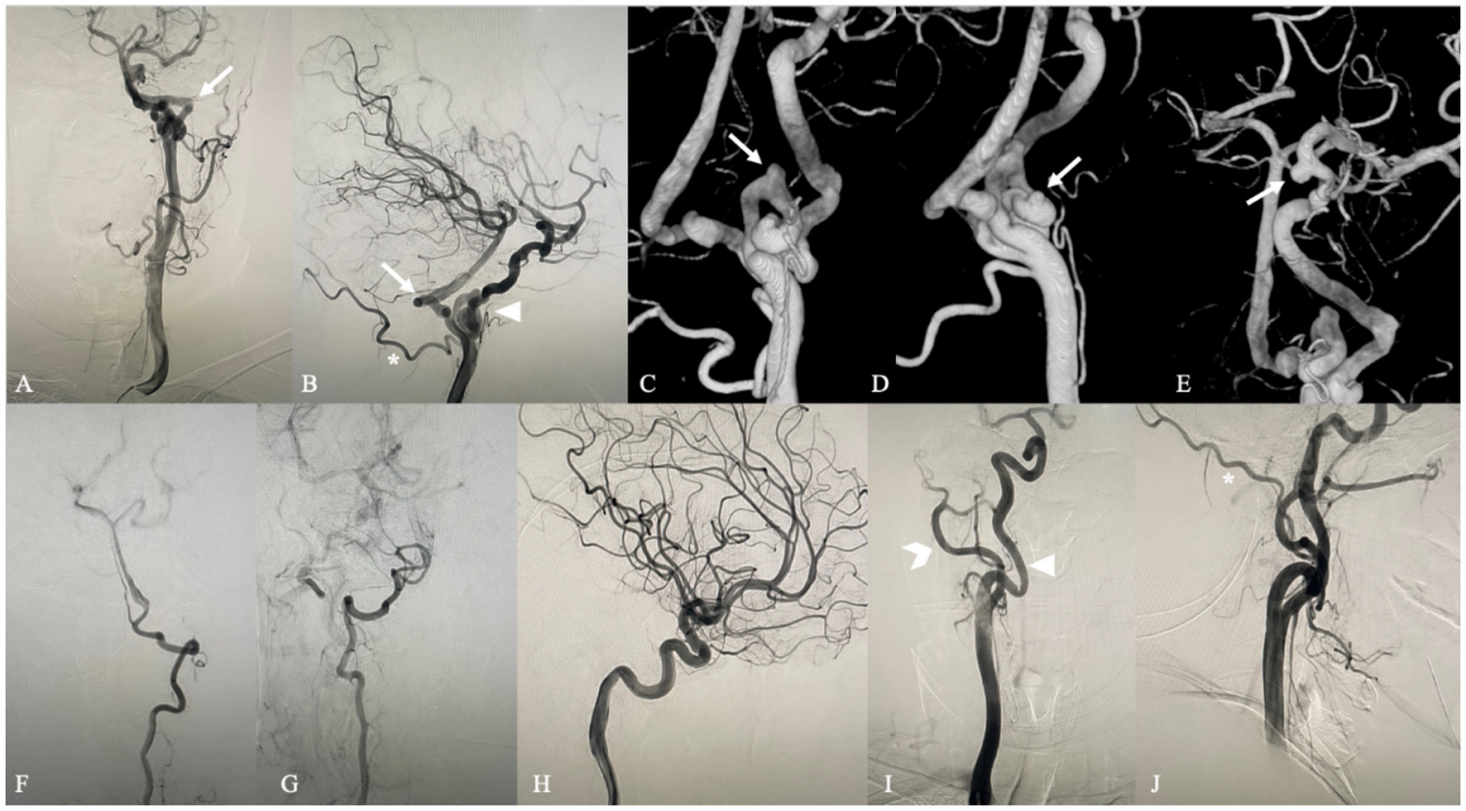
Digital subtraction angiography. Anteroposterior **(A)** and lateral **(B)** left carotid artery angiograms show the PPTA (arrow) originating from the left cervical segment of the ICA and converging to the proximal part of the basilar artery. An extremely coiling segment of the ICA similar to a ball of string exists close to the beginning of PPHA distally (arrow head). The left occipital artery (asterisk) arises from the cervical segment of the ICA rather than the external carotid artery (forked tail) and is close to the beginning of PPHA proximally. 3D DSA shows two aneurysms at the coiling part of the ICA **(C,D)** (arrow) and an aneurysm at the posterior communicating artery segment of the ICA **(E)** (arrow). Anteroposterior left and right vertebral artery angiograms **(F,G)** demonstrate that bilateral vertebral arteries are hypoplastic with a small caliber. Posterior communicating arteries were not visualized bilaterally through left and right lateral carotid artery angiograms **(B,H)**. Anteroposterior **(I)** and lateral **(J)** right carotid artery angiograms show that the right extracranial ICA (arrowhead) is a normal tortuosity type and that the right occipital artery (asterisk) arises from the right ECA (forked tail).

A 66-year-old woman was admitted to our hospital presenting with intermittent headaches, dizziness and numbness of the right eyelid which she had been experiencing for 5 years. The patient had a medical history of hypertension, and her blood pressure was well controlled by regular medication. No positive findings were found in physical or neurological examinations. Magnetic resonance angiography performed in a local hospital reported an aneurysm of the posterior communicating artery segment of the left ICA and a left PPHA (Figure 1) (1). Further digital subtraction angiography (DSA) was performed after admission and showed a PPHA originating from the left cervical segment of the ICA and converging to the proximal part of the basilar artery (Figures 2A,B). An extremely coiling segment of the ICA, such as a ball of string, distally exists close to the beginning of PPHA (Figure 2B; Supplementary Video 1). In addition, two small aneurysms were found at the coiling segment of the ICA, measuring 1.9 × 1.6 mm and 1.4 × 1.1 mm (Figures 2C,D). The aneurysm of the posterior communicating artery segment of the left ICA was also confirmed by DSA and measured 3.8 × 3.4 mm (Figure 2E). Bilateral vertebral arteries are hypoplastic with a small caliber, and the majority of the blood supply to the posterior circulation comes from PPHA (Figures 2F,G). Posterior communicating arteries were not visualized bilaterally (Figures 2B,H). The left occipital artery arises from the cervical segment of the ICA rather than the external carotid artery (ECA) and is close to the beginning of PPHA proximally (Figure 2B). Except for the anomalies mentioned above, the right extracranial ICA is a normal tortuosity type (Figure 2I). However, the right occipital artery arose from the right ECA (Figure 2J). The rupture risk of the aneurysm of the posterior communicating artery segment of the left ICA is considered low because of the volume and regular shape of the aneurysm. The treatment of this aneurysm could not be achieved by endovascular therapy due to the extreme coiling of the ICA. Clipping is the only feasible way to treat aneurysms. Eventually, the patient denied surgery and decided to undergo regular observation of this aneurysm after discharge.

## Discussion

The hindbrain, which begins to develop when the embryo reaches 4–5 mm in length, is supplied by the immature paired longitudinal neural arteries (LNAs). The blood supply to the LNAs derives from carotid–basilar anastomoses from the primitive ICA, including the primitive trigeminal artery (PTA), primitive optic artery (POA), primitive hypoglossal artery (PHA) and primitive proatlantal artery (ProA) (1). If development of the posterior circulation is delayed or abnormal, these anastomoses can be preserved to supply blood to the posterior circulation (12). In four anastomotic branches, PTA is the most common persistent anastomosis, with an incidence of 0.1–0.6%. PHA is the second most common persistent anastomosis and occurs in 0.02–0.1% of the population. ProA is third in incidence and rarely persists into adulthood. POA is the rarest, and there is scarce evidence to prove its existence. Each of them has its own characteristics of origin on ICA, running route and afflux position of posterior circulation to distinguish in adulthood (13). The PPHA originates from the cervical segment of ICA at C1 to C3 level, and runs through the hypoglossal canal to enter the intracranial space along with the twelfth cranial nerve. Subsequently, PPHA joins the basilar artery with or without the absence of vertebral artery. It is assumed that all of the anastomoses are preserved into adulthood due to genetic factors or abnormal factors during embryonic development. Compared with PPHA, morphological anomalies of the extracranial ICA are more common in clinical practice, and the incidence is 10–43% (10). Tortuosity and coiling of the extracranial ICA are also thought to be congenital variations (11). However, to our knowledge, no attention has been given to the association between PPHA (even PTA, POA and ProA) and morphological anomalies of the extracranial ICA.

In this case, PPHA supplied the majority of the blood to the posterior circulation. There was an extremely coiling part of the ICA that was close to the origin of PPHA distally. The tortuous carotid artery convolves like a ball of string, and there has been no similar morphological anomaly of the ICA, either in tortuosity extent or position, that has ever been reported. According to the classification of Weibel and Fields (11), it belongs to the coiling type of ICA morphological anomalies, which are thought to be formed congenitally (11). Since the coiling part of the ICA was so close to the origin of PPHA, we assumed that PPHA and the abnormal coiling of the ICA were associated and that both were influenced by some congenital factors in embryonic development. Moreover, multiple aneurysms were found on the coiling segment and posterior communicating artery segment of the ICA. Aneurysms are common concomitant lesions with PPHA and are thought to be formed by congenital defects of the artery wall or hemodynamics (2–4). It is worth noting that aneurysms on the coiling part of the ICA prove the defect of vasculature of this morphological abnormality.

**Figure 3:**
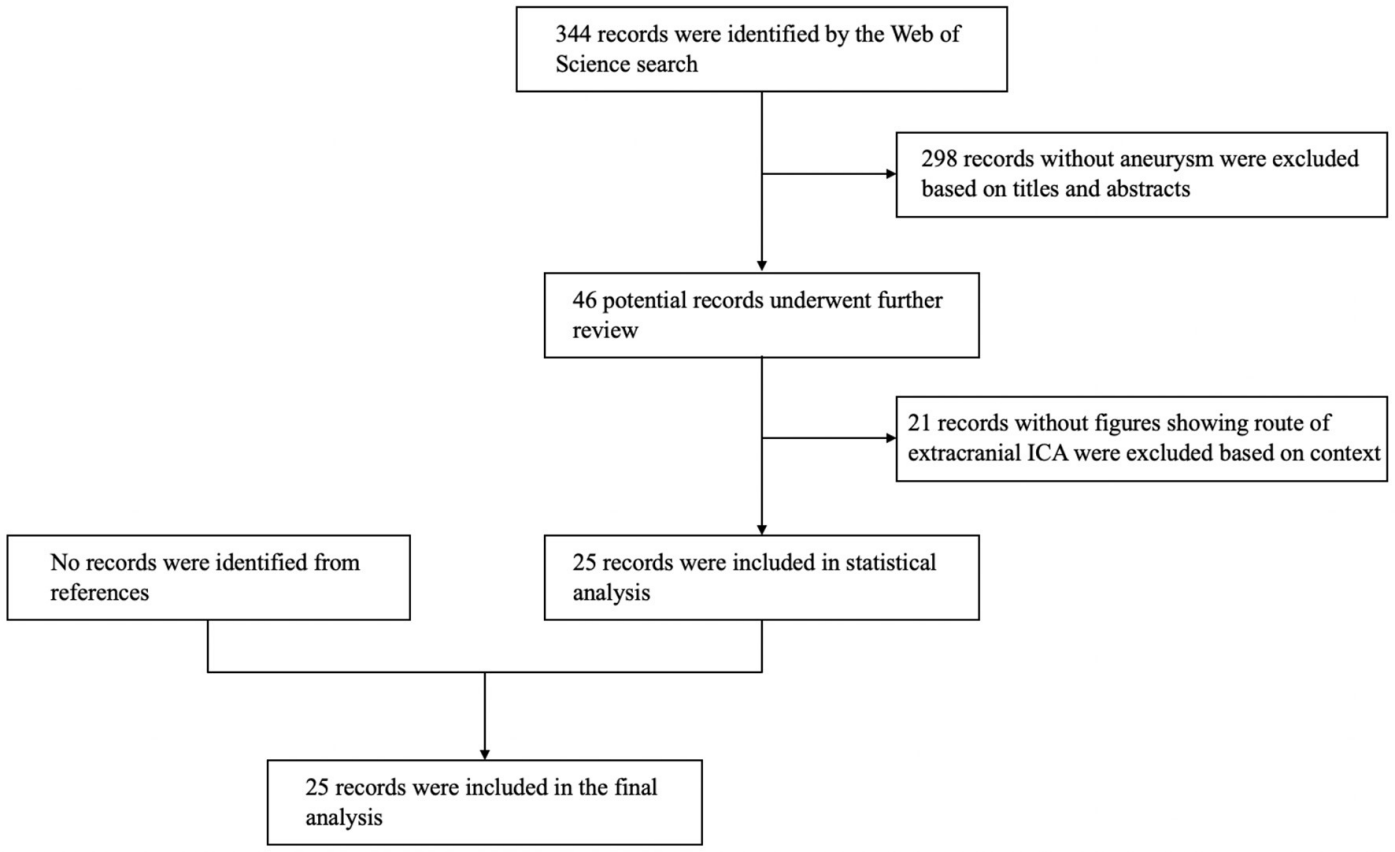
Flow chart of the search strategies.

There is no relevant report or research on concomitant PPHA and morphological anomalies of the extracranial ICA. We conducted a literature review on the articles of concomitant PPHA and aneurysms and paid attention to the morphology of the extracranial ICA. We searched the Web of Science database and used the terms “primitive hypoglossal artery,” “primary hypoglossal artery,” “persistent hypoglossal artery” and “persistent primitive hypoglossal artery.” We extracted articles regarding PPHA with aneurysms and excluded articles that did not have the figures showing the route of extracranial ICA. The resulting flowchart of the search strategies is shown in Figure 3. Twenty-five reports of 28 patients were finally included for statistical analysis. The morphology data of the extracranial ICA of these patients are shown in Table 1 (2, 6, 14–36).

**Table 1:** Summary of 28 cases of persistent primitive hypoglossal artery (PPHA) and aneurysms.

**References**	**Case no**.	**Age/Sex**	**Position of aneurysms**	**Morphology of extracranial ICA (ipsilateral to PPHA)**	**Morphology of extracranial ICA (contralateral to PPHA)**
Zhang et al. (2)	1	73/F	Basilar bifurcation	Tortuosity	NA
Murumkar et al. (14)	2	32/F	Right posterior cerebral artery	Tortuosity	Normal
Kawamura et al. (15)	3	67/F	ICA bifurcation (ipsilateral)	Normal	NA
He et al. (16)	4	43/F	PPHA	Normal	Normal
Haryu et al. (17)	5	45/F	Anterior choroidal artery (ipsilateral)	Tortuosity	NA
Zeng et al. (18)	6	83/F	PPHA	Tortuosity	NA
Tse et al. (19)	7	80/F	PPHA	Tortuosity	NA
Ho et al. (20)	8	74/F	Cervical ICA (ipsilateral)	Tortuosity	Normal
Hopf-Jensen et al. (21)	9	50/F	ICA bifurcation (ipsilateral)	Tortuosity	NA
Kimball et al. (36)	10	42/F	PPHA	Tortuosity	Normal
Uchino et al. (22)	11	70/F	Basilar artery	Tortuosity	Tortuosity
	12	62/F	ACA–anterior communicating artery (contralateral)	Normal	Tortuosity
	13	52/F	Middle cerebral artery (contralateral)	Tortuosity	Tortuosity
Teo et al. (23)	14	52/F	Basilar bifurcation	Tortuosity	NA
			ICA bifurcation (ipsilateral)		
			Middle cerebral aneurysm (contralateral)		
Pavlisa et al. (6)	15	61/F	Anterior inferior cerebellar artery	Tortuosity	NA
Hui et al. (24)	16	61/M	PPHA	Tortuosity	NA
Furtado et al. (25)	17	65/M	Basilar artery	Tortuosity	NA
Baldi et al. (26)	18	70/F	PPHA	Tortuosity	NA
Kobayashi et al. (27)	19	49/F	Posterior inferior cerebellar artery	Tortuosity	NA
Baltsavias et al. (28)	20	46/F	PPHA	Tortuosity	NA
Hatayama et al. (29)	21	71/F	Posterior communicating artery segment of ICA (ipsilateral)	Tortuosity	NA
Murayama et al. (30)	22	59/M	PPHA	Normal	Normal
Waga et al. (31)	23	54/F	PPHA	Tortuosity	NA
			Anterior cerebral artery (contralateral)		
Bongartz et al. (32)	24	28/F	PPHA	Normal	NA
Kodama et al. (33)	25	48/F	Anterior communicating artery	Tortuosity	NA
	26	34/F	PPHA	Tortuosity	NA
Anderson (34)	27	29/F	Basilar bifurcation	Normal	NA
Huber and Rivoir (35)	28	62/F	PPHA	Normal	NA

The average age of the 28 patients was 55.82 ± 15.159 years old. The average age of patients with and without tortuous extracranial ICA is 57.71 ± 14.61 and 50.00 ± 16.49, respectively, and the youngest patient in the groups with and without tortuous extracranial ICA was 32 years old and 28 years old. Eighty-nine percent (25/28) were female. Thirty-one aneurysms in total were found in 28 patients. Sixty-five percent (20/31) were located in the posterior circulation and sixty percent (12/20) were located in PPHA. Of 10 aneurysms in the anterior circulation, 60% (6/10) were located ipsilaterally to the PPHA. Yamamoto et al. (37) review the literature and found 26% of cases of PPHA coexisted with intracranial aneurysms. But selection bias owing to the absence of general population study still makes the association between PPHA and aneurysm controversial. The curvature of carotid artery was also found to be correlated with aneurysm presence in any its intracranial segments (38). From our perspective, the hemodynamic change yielded by PPHA and tortuosity of ICA might be one of the factors accounting for the formation of aneurysm.

Of 28 patients in this analysis, 75% (21/28) had tortuosity of the extracranial ICA ipsilaterally to the PPHA, and this incidence was higher than 10–43% of the normal population (10). Interestingly, of 8 cases which had contralateral carotid angiograms, 37% (3/8) showed tortuosity of the extracranial ICA contralaterally to the PPHA which is similar to the normal incidence. No kinking or coiling existed in these cases. The results indicate that PPHA might be associated with the tortuosity of the ipsilateral extracranial ICA. Since tortuosity of the ICA also comes from congenital factors, PPHA and tortuosity of the ICA are probably both formed by genetic factors or abnormalities in the development of the embryo. However, there is no report of coiling of the ICA, which is also caused by congenital factors, in the review of articles. This might be caused by the two-dimensional observation of figures in the report. Through the statistics, the average age of patients without tortuous extracranial ICA is younger than patients with tortuous extracranial ICA. The age also might be a factor influencing the presence of tortuous extracranial ICA. However, because of the small number of reported cases of these rarely concurrent vascular anomalies, more evidence is needed for verification. Morphological anomalies similar to the extremely coiling ICA in this article have not been found in any report. By means of the literature review, we assume the association of the existence of PPHA and morphological anomalies of the extracranial ICA. Endovascular therapy of the aneurysm is not feasible in this case because of coiling of the ICA. Microsurgical therapy is the only way to treat aneurysms. Considering the association of PPHA and morphological anomalies of the ICA, clinicians need to know that endovascular treatment of aneurysms in patients with PPHA may be faced with a tortuous pathway to the aneurysm.

## Conclusion

To the best of our knowledge, this is the first report of an extremely coiling ICA of such a tortuous nature and at this position. Meanwhile, PPHA and multiple aneurysms are found ipsilaterally. We were inspired by this case and chose to explore the relation between the tortuosity of ICA and the persistence of PHA using a review of the literature. The results of the review show that the tortuosity of the ICA might be associated with PPHA. Since no studies have focused on the association between ICA tortuosity and carotid-basilar anastomoses, including PTA, POA and ProA, more studies are warranted in the future.

## Author contributions

All authors listed have made a substantial, direct, and intellectual contribution to the work and approved it for publication.

## Conflict of interest

The authors declare that the research was conducted in the absence of any commercial or financial relationships that could be construed as a potential conflict of interest.

## Publisher's note

All claims expressed in this article are solely those of the authors and do not necessarily represent those of their affiliated organizations, or those of the publisher, the editors and the reviewers. Any product that may be evaluated in this article, or claim that may be made by its manufacturer, is not guaranteed or endorsed by the publisher.

## Supplementary material

Supplementary Video 1 Rotation imaging of 3D DSA of the left carotid artery.
